# Human Parvoviruses May Affect the Development and Clinical Course of Meningitis and Meningoencephalitis

**DOI:** 10.3390/brainsci10060339

**Published:** 2020-06-03

**Authors:** Anda Vilmane, Anna Terentjeva, Paulius L. Tamosiunas, Normunds Suna, Inga Suna, Rasa Petraityte-Burneikiene, Modra Murovska, Santa Rasa-Dzelzkaleja, Zaiga Nora-Krukle

**Affiliations:** 1 Institute of Microbiology and Virology, Rīga Stradiņš University, 5 Ratsupites St., LV-1067 Riga, Latvia; anna.terentjeva@rsu.lv (A.T.); modra.murovska@rsu.lv (M.M.); santa.rasa-dzelzkaleja@rsu.lv (S.R.-D.); zaiga.nora@rsu.lv (Z.N.-K.); 2 Vilnius University Life Sciences Center Institute of Biotechnology, 7 Sauletekio Al., 10257 Vilnius, Lithuania; paulius.tamosiunas@bti.vu.lt (P.L.T.); rasa.burneikiene@bti.vu.lt (R.P.-B.); 3 Department of Neurology and Neurosurgery, Riga East Clinical University Hospital “Gaiļezers”, 2 Hipokrata St., LV-1038 Riga, Latvia; n.suuna@gmail.com (N.S.); suuna.inga@gmail.com (I.S.)

**Keywords:** human parvovirus B19, human bocaviruses 1–4, human parvovirus 4, meningitis, meningoencephalitis

## Abstract

Meningitis and meningoencephalitis are neurological inflammatory diseases, and although routine diagnostics include testing of a wide range of pathogens, still in many cases, no causative agent is detected. Human parvovirus B19 (B19V), human bocaviruses 1–4 (HBoV1–4), and human parvovirus 4 (hPARV4) are members of the *Parvoviridae* family and are associated with a wide range of clinical manifestations including neurological disorders. The main aim of this study was to determine whether human parvoviruses infection markers are present among patients with meningitis/meningoencephalitis in Latvia as well as to clarify the role of these viruses on the clinical course of the mentioned diseases. Our study revealed HBoV1–4 and B19V genomic sequences in 52.38% and 16.67% of patients, respectively. Furthermore, symptoms such as the presence of a headache and its severity, fatigue, disorientation, and difficulties to concentrate were significantly frequently present in patients with active parvovirus infection in comparison with parvoviruses negative patients, therefore we suggest that HBoV1–4 and B19V infection should be included in the diagnostics to reduce the number of meningitis/meningoencephalitis with unknown/unexplained etiology.

## 1. Introduction

Viral meningitis and meningoencephalitis are medical emergencies that should be recognized early and properly treated. A recent prospective cohort study in the United Kingdom showed that the estimated incidence of viral meningitis was 2.73 per 100,000 adults [1]. In a larger retrospective study from Qatar, the determined incidence of viral infections of the central nervous system (CNS) was 6.4 per 100,000 [2]. Enteroviruses, herpes simplex viruses (HSV), and varicella zoster virus (VZV) are the major causative agents detected, but for a significant number of clinical cases, no etiological agent is revealed [2,3,4].

Human parvovirus B19 (B19V), human bocaviruses 1–4 (HBoV1–4), and human parvovirus 4 (hPARV4) are members of *Parvoviridae*, a family of small (~25 nm) non-enveloped viruses containing a linear single stranded DNA (ssDNA) genome of 5 to 6 kb [5]. B19V, HBoV1–4, and hPARV4 belong to the *Parvovirinae* subfamily and *Erythroparvovirus*, *Bocaparvovirus*, and *Tetraparvovirus* genus, respectively [6]. These parvoviruses are considered as human pathogens and are associated with a wide range of pathologies. For example, B19V causes fifth disease, persistent anemia, transient aplastic crisis, hydrops fetalis, and arthropathy [7,8,9]. HBoV1 is considered as a respiratory pathogen and causes upper and lower respiratory tract diseases in children, but HBoV2–4 are mostly found in stool samples and are associated with gastroenteritis [10]. Both B19V and HBoV1–4 have been linked with neurological disorders as well [11,12,13,14,15,16,17,18,19]. There are few reports in the literature describing the presence of HBoVs DNA in patients with encephalitis and encephalopathy, showing its ability to enter the CNS and its possible role in causing disease [11,12,13,14,15]. B19V infection has been associated with various neurological complications such as encephalitis, meningitis, stroke, neuropathy, status epilepticus, and encephalopathy. While B19V infection is more frequently found among immunocompromised hosts, it can occur in the immunocompetent, apparently healthy children and adults as well [16,17,18,19]. Of the above-mentioned parvoviruses, hPARV4’s role in causing diseases has been least studied and is still ambiguous. Initially, it was considered that hPARV4 infection was present only among intravenous drug abusers, and, in general, hPARV4 viremia appeared to be self-limiting and asymptomatic [20,21]. Although a possible clinical manifestation of hPARV4 infection has been reported, including respiratory or gastrointestinal symptoms, rash, and also encephalitis, the association between hPARV4 and the disease needs to be clarified [20,22,23,24].

This is the first study aiming to simultaneously determine the frequency of B19V, HBoV1–4, and hPARV4 infection markers and to evaluate the involvement of parvoviruses in the etiology and the clinical course of meningitis and meningoencephalitis.

## 2. Materials and Methods

### 2.1. Study Groups

In total, 42 cases of confirmed or unknown etiology of meningitis (*n* = 31; 73.81%) or meningoencephalitis (*n* = 11; 26.19%) were evaluated between June 2014 and October 2018. Of all the patients, 20 (47.6%) were males and 22 (52.4%) were females, with the mean age ± standard deviation (SD) 50 ± 18.2 and 58.9 ± 19.8 years, respectively. In all cases, whole blood and cerebrospinal fluid (CSF) samples were obtained on admission by certified medical personnel accordingly to all safety standards. Information about patient demographics, clinical diagnoses, duration of hospitalization and outcome, symptoms (present or absent)-headache (assessed by the numeric pain scale), increased body temperature (≥37 °C), fatigue, sleepiness, difficulties to concentrate, disorientation, seizures, muscle ache, weight loss, nausea, diarrhea, dizziness, nuchal rigidity, Kernig’s sign, photo- and phonophobia and the main blood test-level of white blood cells (WBC) (10^9^/L), red blood cells (RBC) (10^12^/L), platelets (PLT) (10^9^/L), hemoglobin (Hgb) (g/dL), hematocrit (Hct) (%), C-reactive protein (CRP) (mg/L), and microbiological test results, as well as the information about CSF analysis-pleocytosis (µL), agranulocyte and granulocyte count (%), protein (g/L), and glycose (mmol/L) level were analyzed retrospectively from the clinical data of Riga East Clinical University Hospital (RECUH). All the whole blood and CSF samples were microbiologically tested in the Latvian Centre of Infectious Diseases (LCID) for a group of infectious agents-tick-borne encephalitis virus (TBEV) infection was diagnosed by the presence of anti-TBEV IgM specific antibodies in the cell-free blood plasma using the enzyme-linked immunosorbent assay (ELISA); enteroviruses—by the presence of viral RNA in the CSF using the reverse transcription polymerase chain reaction (PCR); VZV, HSV, cytomegalovirus (CMV), and Epstein–Barr virus (EBV) were diagnosed by the presence of viral DNA in the CSF using nested PCR (nPCR). *Borrelia burgdorferi* (*B.Burgdorferi*) infection was diagnosed by the presence of anti-*B.Burgdorferi* IgM and/or IgG in the cell-free blood plasma using immunoblot. To confirm *B.Burgdorferi* CNS infection, an antibody index was calculated according to the formula (Ig_CSF *B.Burgdorferi*_/Ig_serum *B.Burgdorferi*_)/(Ig_CSF total_/Ig_serum total_) using ELISA (U/mL). The antibody index >1.5 was considered diagnostic for CNS Lyme disease. Carcinomatous meningitis was diagnosed with head magnetic resonance imaging, confirming carcinomatous infiltration of the meninges of the brain or by the presence of cancer cells in the CSF.

Fifty peripheral blood and corresponding cell-free blood plasma samples of apparently healthy blood donors (age and gender matched) were included in the study as a control group. Due to ethical and clinical issues, the collection of CSF samples from healthy persons was not possible.

The study protocol and the use of human biological material were approved by the Ethics Committee of Rīga Stradiņš University (Decisions of the RSU Ethics Committee issued 30 May 2013. and 30 March 2017). Human biological material samples were taken in accordance with the principles of the Declaration of Helsinki. Written informed consent form was obtained from all the individuals included in this study.

### 2.2. Patients Inclusion Criteria

The inclusion criteria for this study were: adult patients admitted to RECUH Neurological Department with ICD-10-CM (International Classification of Diseases, Tenth Revision, Clinical Modification) diagnosis A69.21 (meningitis due to Lyme disease), A69.22 (other neurologic disorders in Lyme disease/meningoencephalitis due to Lyme disease), A84.1 (central European tick-borne encephalitis), B00.3 (herpesviral meningitis), B00.4 (herpesviral encephalitis), B02.0 (zoster encephalitis), B02.1 (zoster meningitis), B27.12 (cytomegaloviral mononucleosis with meningitis), B25.8 (encephalitis due to cytomegalovirus), B27.02 (gammaherpesviral mononucleosis with meningitis), A87.0 (enteroviral meningitis), A85.0 (enteroviral encephalitis), C70.9 (malignant neoplasm of meninges, unspecified), A87.9 (viral meningitis, unspecified), or G03.0 (nonpyogenic meningitis) and pleocytosis >6 µL in CSF. The exclusion criteria were: patients with ICD-10-CM diagnosis G00 (bacterial meningitis, not elsewhere classified), G01 (meningitis in bacterial diseases classified elsewhere), or G04.2 (bacterial meningoencephalitis and meningomyelitis, not elsewhere classified).

### 2.3. Detection of Parvovirus-Specific Antibodies

IgG class antibodies against HBoV1–4 and hPARV4 antigens in all patients and controls cell-free blood plasma samples were measured using the indirect enzyme immunoassay (EIA) format using yeast-generated HBoV1–4 and hPARV4 VP2 virus-like particles as described previously [25,26]. An analogous procedure was used to determine the HBoV1–4 and hPARV4 immunoglobulin concentrations in corresponding CSF samples of 24 patients, only 1:40 sample dilutions were used. The cut-off values were determined ranking the absorbance values and determining the inflection point for each antigen [27,28]. The cut-offs for EIA were 0.447 (HBoV1 IgG), 0.179 (HBoV2–4 IgG), and 0.252 (hPARV4 IgG). The RecomWell Parvovirus B19 IgG test kit (Mikrogen GmbH, Neuried, Germany) was used to examine the patients and control groups’ plasma samples for antibodies against B19V.

### 2.4. Detection of Parvoviruses Genomic Sequences

Total DNA was extracted from peripheral blood, cell-free blood plasma, and CSF samples using a standard phenol-chloroform extraction method. To exclude any possibility of cell DNA contamination, the plasma samples were treated with DNase I (Fermentas, Vilnius, Lithuania) before DNA purification. The quantity of extracted DNA was measured spectrophotometrically and the quality checked by β-globin PCR. The presence of parvovirus genomic sequences in DNA samples was determined using nPCR targeting hPARV4 VP2 gene sequence as described before [29], and the B19V NS1 gene sequence in accordance with Barah et al., 2001, respectively [30]. The presence of HBoV1–4 genomic sequences was determined using primers targeting the HBoV1 NS1 gene sequence and the HBoV1–4 VP1/2 region, respectively, as described before [31,32]. HBoVs, B19V, and hPARV4 positive DNA samples (previously proved by sequencing) were used as positive controls in corresponding PCR. The negative (molecular grade water) control was included after each five samples in each PCR to exclude contamination. All PCR products were analyzed electrophoretically in agarose gel.

### 2.5. Data Analysis

The statistical analysis was performed for continuous data expressed as the mean value and SD or median and interquartile range (IQR). The categorical data were expressed as counts and percentages. To test whether the collected numerical data were normally distributed, the Shapiro–Wilk normality test was applied. Comparisons between the patients and control group as well as subgroups were made using nonparametric Mann–Whitney U test or Kruskal–Wallis test and cross-tabulations (Pearson Chi-square test and Fisher’s exact tests). A *p*-value < 0.05 was considered statistically significant.

All calculations and statistical analysis were performed using the program Prism 7.04 (GraphPad, San Diego, CA, USA).

## 3. Results

### 3.1. Presence of Parvovirus-Specific IgG Class Antibodies

In total, 26/42 (61.9%) patients were HBoV1–4 seropositive and for three patients, HBoV1–4 IgG class antibodies were detected also in the CSF sample. In the control group, HBoV1–4 IgG class antibodies were detected in 29/50 (58.0%) plasma samples. No statistical difference was observed between the patients and control group (*p* > 0.9999). hPARV4 specific IgG class antibodies were detected only in one (2.38%) patient plasma sample and in 5/50 (10.0%) control group plasma samples (*p* = 0.2141), and B19V IgG class antibodies—in 35/42 (83.33%) patients and in 37/50 (74.0%) controls plasma samples (*p* = 0.3194). In addition, 5/42 (11.9%) patients and 4/50 (8.0%) controls plasma samples were negative for all the analyzed parvoviruses-specific IgG class antibodies (*p* = 0.7270). The presence of virus-specific IgG class antibodies is summarized in Table 1.

### 3.2. Presence of Parvoviruses Genomic Sequences

HBoV1–4 DNA was significantly more often found in the patients—22/42 (52.38%) compared to the controls—14/50 (28.0%) (*p* = 0.0199). Most often, HBoV1–4 DNA was present in the patients’ blood (*n* = 17), followed by the cell-free blood plasma (*n* = 8) and CSF (*n* = 7) DNA. In the control group, 12 blood and two plasma samples were HBoV1–4 positive. HBoV1–4 genomic sequences significantly more often were found in the patients’ plasma samples compared with the control group (*p* = 0.0394). B19V genomic sequence was detected in 7/42 (16.67%) patients and 3/50 (6.0%) controls (*p* = 0.1768). Most often, B19V was detected in the patients’ blood (*n* = 5), followed by CSF (*n* = 2) and plasma (*n* = 1), but in the control group, in two blood and one plasma samples. The simultaneous presence of HBoV1–4 and B19V genomic sequences were detected only in one patient—HBoV1–4 genomic sequence was detected in the patients’ CSF sample, but B19V in DNA extracted from the cell-free blood plasma. The presence of the hPARV4 genomic sequence was not found in any of the patients or controls DNA samples. Distribution of the simultaneous presence of HBoV1–4 and B19V genomic sequences among different sample types is summarized in Table 2. The presence of viral genomic sequences in DNA isolated from the cell-free blood plasma and/or CSF samples was evaluated as a marker of active viral infection, but the presence in DNA isolated from the whole blood sample—a persistent viral infection. In addition, 15/22 (63.64%) HboV1–4 positive patients had meningitis and 7/22 (31.82%)—meningoencephalitis. Furthermore, 6/7 (71.43%) B19V positive patients had meningitis and one had meningoencephalitis. One patient with simultaneous HBoV1–4 and B19V infection had meningitis.

### 3.3. Presence of Pathogens Determined in the Clinics

In 22/42 (52.38%) cases, a causative infectious agent of the disease was detected in the LCID. VZV was the most frequently detected etiological agent accounting for 11/42 (26.19%) cases, followed by TBEV (*n* = 3; 7.14%), enteroviruses (*n* = 3; 7.14%), *B.Burgdorferi* (*n* = 3; 7.14%), and EBV (*n* = 2; 4.76%). CMV and HSV were not present in any of cases. The presence of HBoV1–4 and B19V genomic sequences together with other infectious agents is summarized in Table 3. In 8/42 (19.05%) cases, HBoV1–4 was the only infectious agent detected, and B19V alone was detected in 3/42 (7.14%) cases (*p* = 0.1944). Furthermore, in two out of eight HBoV1–4 mono-infections, a viral infection marker was detected in the patients CSF sample, but among three B19V mono-infections in one case—in CSF.

### 3.4. Patients Clinical Characteristics

In 35/42 (83.3%) cases presented with lymphocytic CSF (>50% lymphocytes), they were classified as lymphocytic meningitis. Fatigue was the most common reported symptom (81.0%), followed by headache (76.2%), with the median severity score 9 (IQR 7.00–9.25), fever (69%) with the median temperature 38 °C (IQR 37.8–38.6), sleepiness (57.1%), dizziness (50.0%), weight loss (45.2%), difficulties to concentrate (45.2%), disorientation (38.1%), photophobia (23.8%), phonophobia (21.4%), myalgia (19.0%), nausea (14.3%), seizures (7.1%), and diarrhea (7.1%). The most common meningeal signs were nuchal rigidity (66.7%) and the Kernig’s sign (19.0%). The CSF analysis and blood test results are shown in Supplement Tables S1 and S2, respectively.

In order to evaluate if there were some clinical differences in the case of active parvovirus infection and active infection associated with other infectious agents, patients were divided into two groups and compared mutually: 1st group—patients with active parvovirus infection (*n* = 15) and 2nd group —patients with active infection associated with other infectious agents detected in the clinics (*n* = 12). Active parvovirus infection was defined as the presence of viral genomic sequences in DNA isolated from the patients CSF and/or cell-free blood plasma. All clinical features compared between two groups are summarized in Supplement Table S3. The results revealed that headaches (*p* = 0.0142), headache severity score (*p* = 0.0142), fatigue (*p* = 0.0299), disorientation (*p* = 0.0043), and difficulties to concentrate (*p* = 0.0316) were significantly more often present in the patients with active parvovirus infection in comparison to the patients with active infection associated with other pathogens. The presence of myalgia (*p* = 0.0553) had a tendency to be more often in the patients with active parvovirus infection (Table 4).

## 4. Discussion

This study was set up in order to determine the presence of B19V, HBoV1–4, and hPARV4 infection markers-viral DNA and virus-specific IgG class antibodies, and to evaluate the role of parvoviruses in the inflammatory neurological disorders as meningitis and meningoencephalitis (the rate of meningitis:meningoencephalitis in this study was 2.82:1).

To examine whether parvoviruses have some effect on clinical features of the above-mentioned disorders, we compared the patients with active parvovirus infection and those with active infection associated with other infectious agents. This study demonstrates the frequency of HBoV1–4, B19V, and hPARV4 specific IgG class antibodies among the adult patients with meningitis or meningoencephalitis as well as apparently healthy individuals (control group) in Latvia. The B19V seropositivity among the patients and controls is 83.33% and 74.0%, respectively. The results are similar with those from the other studies where the seroprevalence of B19V among the adult individuals varies from 40% to 80% [33,34,35,36].

In literature, it is stated that the HBoV seroprevalence is age-related with an average of 76.6% in children and 96% in adults [28,37]. However, it should be noted that these data were obtained using the EIA format without competition. In our study, the HBoV1–4 seropositivity is similar in both the patients and control group accounting for 61.9% and 58.0%, respectively. In previous studies, where the HBoV1–4 seropositivity was determined using the competitive EIA format as we have used, similar results were observed—in the study done by Kantola et al., the seropositivity among Finnish and Pakistan adults was 64% and 53%, respectively [28]. In addition, in the study done in Lithuania, the HBoV seropositivity after the competition assay among the patients with respiratory tract diseases was 44.2% [25].

The data from the previous studies show that the seroprevalence of hPARV4 varies geographically. The highest hPARV4 seropositivity is observed in sub-Saharan Africa, ranging from 20% to 37% [38]. In Europe, the hPARV4 seropositivity varies among different studies—4.76% in the United Kingdom and 0% in both Finland and Denmark, respectively [29,39,40]. Relatively high hPARV4 seropositivity has been dated in Lithuania—using the same in-hose EIA method as we used, the frequency of hPARV4-speciffic IgGs in low-risk population was 9.4% [26]. In our study, the presence of hPARV4-specific IgG class antibodies among the patients is relatively low—2.38%, but higher in the control group, accounting for 10%. In order to distinguish between acute and past viral infection among the patients and control group, the presence of parvovirus-specific IgM class antibodies should be determined in the future. This is the first study showing the presence of HBoV1–4 DNA in adult patients with neurological disorders as well as in apparently healthy blood donors in Latvia. The HBoV1–4 DNA significantly more often is detected in the patients (52.38%) compared to the control group (28.0%) (*p* = 0.0199), showing its possible association with the mentioned pathologies. Our HBoV1–4 DNA findings (52.38%) are higher than on average in the world, which varies from 2% to 19% in the patients with respiratory tract diseases [10] and is relatively low in the patients with neurological disorders. For example, the study in Sri-Lanka demonstrated that 2.15% of CSF samples of the patients with encephalitis, including two adults and three children, were HBoVs DNA positive [13]. In the research done in Bangladesh, HBoVs DNA was found in 5.8% of children with encephalitis [12]. It was reported that among the patients with respiratory tract diseases, 0.35% had encephalopathy as a complication of HBoV infection [14]. The presence of HBoV genomic sequences in blood, plasma, or serum samples of apparently healthy adults had been reported before, supporting the hypothesis that HBoVs infection can be asymptomatic. For example, in the research carried out in China, 9.06% of healthy plasma donors were HBoV DNA positive. In the study carried out by an Italian research group, HBoV DNA was found in 5.51% of serum samples of apparently healthy blood donors, but in the study done in Saudi Arabia, 7.0% of blood donors showed HBoV viremia [41,42,43].

The B19V genomic sequence is detected in 16.67% of the patients and 6.0% of the controls. In the latest literature data, the presence of the B19V DNA among patients with different diseases varies—1.13% in the patients presenting with fever with or without rash, arthropathy, and chronic renal disease, and 5.1% in the patients with respiratory diseases [44,45]. In the research done in Iran, B19V DNA was found in 1.2% of blood donors [46].

Although the hPARV4 genomic sequences have been found in healthy individuals [47,48], as well as in two children with an acute encephalitis syndrome of an unknown etiology [49], we do not detect hPARV4 DNA either in the patients or in the control group, showing that this virus is not present among healthy individuals and the patients with inflammatory neurological diseases in Latvia.

Among all patients, parvovirus mono-infection was detected in 11 (26.19%) cases from which eight cases were HBoV1–4 mono-infection and three cases—B19V. Furthermore, in five of 11 mono-infection cases, active parvovirus infection was determined in four cases for HBoV1–4 and in one case for B19V, from which in two cases, HBoV1–4 and in one case, B19V was detected in CSF, suggesting that HBoV1–4 and B19V could be involved in the disease etiology. In addition, in the literature, HBoV1–4 and B19V mono-infection in a case of neurological disorders has been considered as a causative agent [11,12,13,14,19,30,50].

Overall, the information in the literature is scarce about clinical features of the patients with neurological diseases in the case of parvovirus infection. In this particular study, the clinical signs such as the presence of a headache and its severity, fatigue, disorientation, and difficulties to concentrate are significantly frequently present in the patients with active parvovirus infection in comparison with those with active infection associated with other infectious agents (*p* = 0.0142, *p* = 0.0142, *p* = 0.0299, *p* = 0.0043, and *p* = 0.0316, respectively). Whereas myalgia (*p* = 0.0553) has a tendency to be more frequent in the patients with active parvovirus infection, indicating that parvoviruses have an impact on the course of the disease. In some studies, the presence of a headache or disorientation is mentioned as clinical manifestations for the patients with B19V or HBoV1–4 infection [13,51,52]. It is known that B19V causes acute anemia in immunocompromised individuals and pure red blood cell aplasia, which are conditions characterized by an abnormal formation of red blood cells [5,53]. Furthermore, it is dated that B19V infection is associated with a significant decrease in Hgb level among adults and children [54,55,56]. However, in this study, there are no differences observed in the Hgb level and other parameters of blood and the CSF tests such as WBC and RBC count, pleocytosis, glucose level etc., between the patients with active parvovirus infection and the patients with active infection caused by other infectious agents, showing that parvovirus infection does not cause specific changes in blood and CSF composition among the patients with meningitis or meningoencephalitis.

This study shows that HBoV1–4, B19V, and hPARV4 infection markers are present among the apparently healthy adults in Latvia. Based on the results of our study, HBoV1–4 and B19V may have a triggering role in the meningitis/meningoencephalitis development and also may affect the clinical course of the disease, therefore, it would be useful to include these viral infections in laboratory diagnostics to reduce the number of meningitis/meningoencephalitis of unknown/unexplained etiology.

## Figures and Tables

**Table 1:** The presence of parvovirus-specific IgG class antibodies in the patients and control group.

	Patients (*n* = 42)	Control Group (*n* = 50)	*p* Value
	*n* (%)		
**Total B19V IgG**	35 (83.33%)	37 (74.0%)	0.3194
**Total HBoV1–4 IgG**	26 (61.9%)	29 (58.0%)	>0.9999
**Total hPARV4 IgG**	1 (2.38%)	5 (10.0%)	0.2141
**Without IgGs**	5 (11.9%)	4 (8.0%)	0.7270
**B19V IgG only**	11 (26.19%)	15 (30.0%)	0.8170
**HBoV 1–4 IgG only**	2 (4.76%)	8 (16.0%)	0.1035
**hPARV4 IgG only**	0 (0%)	1 (2.0%)	>0.9999
**B19V + HBoV1–4 IgG**	23 (54.76%)	18 (36.0%)	0.0929
**B19V + hPARV4 IgG**	0 (0%)	1 (2.0%)	>0.9999
**B19V + HBoV1–4 + hPARV4 IgG**	1 (2.38%)	3 (6.0%)	0.6225

*n*—number of cases; B19V—human parvovirus B19; HBoV1–4—human bocaviruses 1 to 4; hPARV4—human parvovirus 4; IgG—immunoglobulin G.

**Table 2:** The presence of human bocaviruses 1-4 (HBoV1–4) and parvovirus B19 (B19V) genomic sequences among patients and control group.

DNA Biological Material	Patients (*n* = 42)	Control Group (*n* = 50)
	HBoV1–4 Positivity (*n*)	
Blood ^b^	9	12
Plasma ^a^	3	2
CSF ^a^	2	N/A
Blood + plasma ^a^	3	0
Blood + CSF ^a^	3	N/A
Blood + plasma + CSF ^a^	2	N/A
**Total**	**22**	**14**
	**B19V Positivity (*n*)**	
Blood ^b^	4	2
Plasma ^a^	1	1
CSF ^a^	1	N/A
Blood + CSF ^a^	1	N/A
**Total**	**7**	**3**

*n*—number of cases; DNA—deoxyribonucleic acid; HBoV1–4—human bocavirus 1 to 4; B19V—human parvovirus B19; CSF—cerebrospinal fluid; N/A—not applicable; ^a^—active viral infection; ^b^—persistent viral infection.

**Table 3:** The presence of HBoV1–4 and B19V genomic sequences together with markers of other infectious agents among patients.

	Infectious Agents *n*					
	VZV	EBV	*B.Bburgdorferi*	TBEV	Enteroviruses	Not Identified
Parvoviruses positive patients (*n* = 28)	7	2	*2*	3	3	11
Parvoviruses negative patients (*n* = 14)	4	0	1	0	0	9
**Total**	**11**	**2**	**3**	**3**	**3**	**20**

*n*—number of cases; VZV—varicella zoster virus; EBV—Epstein-Barr virus; *B.Burgdorferi*—*Borrelia burgdorferi*; TBEV—tick-borne encephalitis virus.

**Table 4:** Clinical features of the study population by cause of active infection.

	Patients with Active Parvovirus Infection (*n* = 15)	Patients with Other Active Viral Infection (*n* = 12)	*p* Value
Temperature, °C	38.0 (37.4–38.3)	38.0 (37.5–38.1)	0.2848
Headache	15 (100%)	8 (66.7%)	0.0142 *
Headache severity score	9.00 (7.50–9.50)	7.50 (6.25–9.00)	0.0142 *
Fatigue	14 (93.3%)	7 (58.3%)	0.0299 *
Disorientation	9 (60.0%)	1 (8.3%)	0.0043 *
Difficulties to concentrate	10 (66.7%)	32 (25.0%)	0.0316 *
Nuchal rigidity	11 (73.3%)	6 (54.5%)	0.3395
Kernig’s sign	5 (33.3%)	1 (9.1%)	0.1593
Photophobia	5 (33.3%)	2 (16.7%)	0.3451
Phonophobia	5 (33.3%)	2 (16.7%)	0.3451
Myalgia	4 (26.7%)	0 (0.0%)	0.0553

Data are the median (interquartile range) for continuous data and *n* (%) evaluable for categorical data. *n*—number of cases; *—statistically significant.

## Supplementary Materials

The following are available online at https://www.mdpi.com/2076-3425/10/6/339/s1, Table S1: the CSF analysis results of the study population, Table S2: The blood test results of the study population, Table S3: The clinical features of the study population by cause of active infection.

## References

[1] McGill F., Griffiths M.J., Bonnett L.J., Geretti A.M., Michael B.D., Beeching N.J., McKee D., Scarlett P., Hart I.J., Mutton K.J. (2018). Incidence, aetiology, and sequelae of viral meningitis in UK adults: A multicentre prospective observational cohort study. Lancet Infect. Dis..

[2] Ben Abid F., Abukhattab M., Ghazouani H., Khalil O., Gohar A., Al Soub H., Al Maslamani M., Al Khal A., Al Masalamani E., Al Dhahry S. (2018). Epidemiology and clinical outcomes of viral central nervous system infections. Int. J. Infect. Dis..

[3] De Ory F., Avellón A., Echevarría J.E., Sánchez-Seco M.P., Trallero G., Cabrerizo M., Casas I., Pozo F., Fedele G., Vicente D. (2013). Viral infections of the central nervous system in Spain: A prospective study. J. Med. Virol..

[4] Kupila L., Vuorinen T., Vainionpää R., Hukkanen V., Marttila R.J., Kotilainen P. (2006). Etiology of aseptic meningitis and encephalitis in an adult population. Neurology.

[5] Qiu J., Söderlund-Venermo M., Young N.S. (2017). Human parvoviruses. Clin. Microbiol. Rev..

[6] Cotmore S.F., Agbandje-McKenna M., Chiorini J.A., Mukha D.V., Pintel D.J., Qiu J., Soderlund-Venermo M., Tattersall P., Tijssen P., Gatherer D. (2014). The family Parvoviridae. Arch. Virol..

[7] Heegaard E.D., Brown K.E. (2002). Human Parvovirus B19. Clin. Microbiol. Rev..

[8] Young N.S., Brown K.E. (2004). Parvovirus B19. N. Engl. J. Med..

[9] Brown K.E., Young N.S. (1997). Parvovirus B19 in human disease. Annu. Rev. Med..

[10] Jartti T., Hedman K., Jartti L., Ruuskanen O., Allander T., Söderlund-Venermo M. (2012). Human bocavirus-the first 5 years. Rev. Med. Virol..

[11] Mitui M.T., Tabib S.M., Matsumoto T., Khanam W., Ahmed S., Mori D., Akhter N., Yamada K., Kabir L., Nishizono A. (2012). Detection of human bocavirus in the cerebrospinal fluid of children with encephalitis. Clin. Infect. Dis..

[12] Yu J.M., Chen Q.Q., Hao Y.X., Yu T., Zeng S.Z., Wu X.B., Zhang B., Duan Z.J. (2013). Identification of human bocaviruses in the cerebrospinal fluid of children hospitalized with encephalitis in China. J. Clin. Virol..

[13] Mori D., Ranawaka U., Yamada K., Rajindrajith S., Miya K., Perera H.K., Matsumoto T., Dassanayake M., Mitui M.T., Mori H. (2013). Human bocavirus in patients with encephalitis, Sri Lanka, 2009–2010. Emerg. Infect. Dis..

[14] Akturk H., Sık G., Salman N., Sutcu M., Tatli B., Ciblak M.A., Erol O.B., Torun S.H., Citak A., Somer A. (2015). Atypical presentation of human bocavirus: Severe respiratory tract infection complicated with encephalopathy. J. Med. Virol..

[15] Ergul A.B., Altug U., Aydin K., Guven A.S., Altuner Torun Y. (2017). Acute necrotizing encephalopathy causing human bocavirus. Neuroradiol. J..

[16] Douvoyiannis M., Litman N., Goldman D.L. (2009). Neurologic manifestations associated with parvovirus B19 infection. Clin. Infect. Dis..

[17] Barah F., Vallely P., Cleator G.M., Kerr J.R. (2003). Neurological manifestations of human parvovirus B19 infection. Rev. Med. Virol..

[18] Barah F., Whiteside S., Batista S., Morris J. (2014). Neurological aspects of human parvovirus B19 infection: A systematic review. Rev. Med. Virol..

[19] Watanabe T., Kawashima H. (2015). Acute encephalitis and encephalopathy associated with human parvovirus B19 infection in children. World J. Clin. Pediatrics.

[20] Jones M.S., Kapoor A., Lukashov V.V., Simmonds P., Hecht F., Delwart E. (2005). New DNA viruses identified in patients with acute viral infection syndrome. J. Virol..

[21] Panning M., Kobbe R., Vollbach S., Drexler J.F., Adjei S., Adjei O., Drosten C., May J., Eis-Hübingercorresponding A.M. (2010). Novel human parvovirus 4 genotype 3 in infants, Ghana. Emerg. Infect. Dis..

[22] Drexler J.F., Reber U., Muth D., Herzog P., Annan A., Ebach F., Sarpong N., Acquah S., Adlkofer J., Adu-Sarkodie Y. (2012). Human Parvovirus 4 in nasal and fecal specimens from children, Ghana. Emerg. Infect. Dis..

[23] Sharp C.P., Lail A., Donfield S., Gomperts E.D., Simmonds P. (2012). Virologic and clinical features of primary infection with human parvovirus 4 in subjects with hemophilia: Frequent transmission by virally inactivated clotting factor concentrates. Transfusion.

[24] Benjamin L.A., Lewthwaite P., Vasanthapuram R., Zhao G., Sharp C., Simmonds P., Wang D., Solomon T. (2011). Human parvovirus 4 as potential cause of encephalitis in children, India. Emerg. Infect. Dis..

[25] Tamošiūnas P.L., Petraitytė-Burneikienė R., Bulavaitė A., Marcinkevičiūtė K., Simutis K., Lasickienė R., Firantienė R., Ėmužytė R., Žvirblienė A., Sasnauskas K. (2016). Yeast-generated virus-like particles as antigens for detection of human bocavirus 1–4 specific antibodies in human serum. Appl. Microbiol. Biotechnol..

[26] Tamošiunas P.L., Simutis K., Kodze I., Firantiene R., Emužyte R., Petraityte-Burneikiene R., Žvirbliene A., Sasnauskas K. (2013). Production of human parvovirus 4 VP2 virus-like particles in yeast and their evaluation as an antigen for detection of virus-specific antibodies in human serum. Intervirology.

[27] Kean J.M., Rao S., Wang M., Garcea R.L. (2009). Seroepidemiology of human Polyomaviruses. PLoS Pathog..

[28] Kantola K., Hedman L., Arthur J., Alibeto A., Delwart E., Jartti T., Ruuskanen O., Hedman K., Soderlund-Venermo M. (2011). Seroepidemiology of human bocaviruses 1-4. J. Infect. Dis..

[29] Maple P.A., Beard S., Parry R.P., Brown K.E. (2013). Testing UK blood donors for exposure to human parvovirus 4 using a time-resolved fluorescence immunoassay to screen sera and Western blot to confirm reactive samples. Transfusion.

[30] Barah F., Vallely P.J., Chiswick M.L., Cleator G.M., Kerr J.R. (2001). Association of human parvovirus B19 infection with acute meningoencephalitis. Lancet.

[31] Sloots T.P., McErlean P., Speicher D.J., Arden K.E., Nissen M.D., Mackay I.M. (2006). Evidence of human coronavirus HKU1 and human bocavirus in Australian children. J. Clin. Virol..

[32] Kapoor A., Simmonds P., Slikas B., Li L., Bodhidatta L., Sethabutr O., Triki H., Bahri O., Oderinde B., Baba M. (2010). Human bocaviruses are highly diverse, dispersed, recombination prone, and prevalent enteric infections. J. Infect. Dis..

[33] Röhrer C., Gärtner B., Sauerbrei A., Böhm S., Hottenträger B., Raab U., Thierfelder W., Wutzler P., Modrow S. (2008). Seroprevalence of parvovirus B19 in the German population. Epidemiol. Infect..

[34] Mossong J., Hens N., Friederichs V., Davidkin I., Broman M., Litwinska B., Siennicka J., Trzcinska A., van Damme P., Beutels P. (2008). Parvovirus B19 infection in five European countries: Seroepidemiology, force of infection and maternal risk of infection. Epidemiol. Infect..

[35] Ihara T., Furusyo N., Hayashi T., Toyoda K., Murata M., Hayashi J. (2013). A population-based epidemiological survey of human parvovirus B19 infection: A project of the Kyushu and Okinawa Population Study (KOPS). Arch. Virol..

[36] Adam O., Makkawi T., Reber U., Kirberg H., Eis-Hübinger A.M. (2015). The seroprevalence of parvovirus B19 infection in pregnant women in Sudan. Epidemiol. Infect..

[37] Hustedt J.W., Christie C., Hustedt M.M., Esposito D., Vazquez M. (2012). Seroepidemiology of human bocavirus infection in Jamaica. PLoS ONE.

[38] Sharp C.P., Vermeulen M., Nébié Y., Djoko C.F., LeBreton M., Tamoufe U., Rimoin A.W., Kayembe P.K., Carr J.K., Servant-Delmas A. (2010). Changing epidemiology of human parvovirus 4 infection in sub-Saharan Africa. Emerg. Infect. Dis..

[39] Lahtinen A., Kivelä P., Hedman L., Kumar A., Kantele A., Lappalainen M., Liitsola K., Ristola M., Delwart E., Sharp C. (2011). Serodiagnosis of primary infections with human parvovirus 4, Finland. Emerg. Infect. Dis..

[40] Von Linstow M.L., Rosenfeldt V., Lindberg E., Jensen L., Hedman L., Li X., Väisänen E., Hedman K., Norja P. (2015). Absence of novel human parvovirus (PARV4) in Danish mothers and children. J. Clin. Virol..

[41] Li H., He M., Zeng P., Gao Z., Bian G., Yang C., Li W. (2015). The genomic and seroprevalence of human bocavirus in healthy Chinese plasma donors and plasma derivatives. Transfusion.

[42] Bonvicini F., Manaresi E., Gentilomi G.A., Di Furio F., Zerbini M., Musiani M., Gallinella G. (2011). Evidence of human bocavirus viremia in healthy blood donors. Diagn. Microbiol. Infect. Dis..

[43] Abdel-Moneim A.S., Mahfouz M.E., Zytouni D.M. (2018). Detection of human bocavirus in Saudi healthy blood donors. PLoS ONE.

[44] Vadivel K., Mageshbabu R., Sankar S., Jain A., Perumal V., Srikanth P., Ranjan G.A., Nair A., Simoes E.A.F., Nandagopal B. (2018). Detection of parvovirus B19 in selected high-risk patient groups & their phylogenetic & selection analysis. Indian J. Med. Res..

[45] Tavakoli A., Monavari S.H., Mollaei H., Bokharaei-Salim F., Esghaei M., Keyvani H., Ghaffari H. (2018). Frequency of human Parvovirus B19 among patients with respiratory infection in Iran. Med. J. Islamic Repub. Iran.

[46] Zadsar M., Aghakhani A., Banifazl M., Kazemimanesh M., Tabatabaei Yazdi S.M., Mamishi S., Bavand A., Sadat Larijani M., Ramezani A. (2018). Seroprevalence, molecular epidemiology and quantitation of parvovirus B19 DNA levels in Iranian blood donors. J. Med. Virol..

[47] Fryer J.F., Delwart E., Hecht F.M., Bernardin F., Jones M.S., Shah N., Baylis S.A. (2007). Frequent detection of the parvoviruses, PARV4 and PARV5, in plasma from blood donors and symptomatic individuals. Transfusion.

[48] Amirahmadi F., Sarvari J., Hosseini S.Y., Pirbonyeh N., Gorzin A.A. (2017). Frequency of human parvovirus 4 (PARV4) viremia among HBV-infected patients and healthy donors in Shiraz, Iran. Turk. J. Med. Sci..

[49] Arankalle V.A., Srivastava N., Kushwaha K.P., Sen A., Ramdasi A.Y., Patel P.A., Kuthe S., Haldipur B., Sakpal G.N., Lole K.S. (2019). Detection of human parvovirus 4 DNA in the patients with acute encephalitis syndrome during seasonal outbreaks of the disease in Gorakhpur, India. Emerg. Microbes Infect..

[50] Cassinotti P., Schultze D., Schlageter P., Chevili S., Siegl G. (1993). Persistent human parvovirus B19 infection following an acute infection with meningitis in an immunocompetent patient. Eur. J. Clin. Microbiol. Infect. Dis..

[51] Pereira A.C., Barros R.A., Do Nascimento J.P., De Oliveira S.A. (2001). Two family members with a syndrome of headache and rash caused by human parvovirus B19. Braz. J. Infect. Dis..

[52] Nolan R.C., Chidlow G., French M.A. (2003). Parvovirus B19 encephalitis presenting as immune restoration disease after highly active antiretroviral therapy for human immunodeficiency virus infection. Clin. Infect. Dis..

[53] Robert T., Means J. (2016). Pure red cell aplasia. Blood.

[54] Wildig J., Cossart Y., Peshu N., Gicheru N., Tuju J., Williams T.N., Newton C.R. (2010). Parvovirus B19 infection and severe anaemia in Kenyan children: A retrospective case control study. BMC Infect. Dis..

[55] Gasim G.I., Eltayeb R., Elhassan E.M., Haggaz A.D., Rayis D.A., Adam I. (2016). Human parvovirus B19 and low hemoglobin levels in pregnant Sudanese women. Int. J. Gynaecol. Obstet..

[56] Tizeba Y.A., Mirambo M.M., Kayange N., Mhada T., Ambrose E.E., Smart L.R., Mshana S.E. (2018). Parvovirus B19 is associated with a significant decrease in Hemoglobin level among children <5 Years of Age with Anemia in Northwestern Tanzania. J. Trop. Pediatrics.

